# Epidemiology of thymomas and thymic carcinomas in the United States and Germany, 1999-2019

**DOI:** 10.3389/fonc.2023.1308989

**Published:** 2024-01-09

**Authors:** Tiemo Sven Gerber, Stephanie Strobl, Alexander Marx, Wilfried Roth, Stefan Porubsky

**Affiliations:** ^1^ Institute of Pathology, University Medical Center of the Johannes Gutenberg-University Mainz, Mainz, Germany; ^2^ Institute of Pathology, University Medical Center Göttingen, Göttingen, Germany

**Keywords:** thymic epithelial tumors, thymoma, thymic cancer, incidence rate, survival, epidemiology

## Abstract

**Introduction:**

Mediastinal tumors, particularly non-neuroendocrine thymic epithelial tumors (TET) are relatively uncommon, posing challenges for extensive epidemiological studies. This study presents a comprehensive analysis of these tumors in the United States (US) and Germany (GER) from 1999 to 2019.

**Methods:**

Patients aged 0-19 (n=478) and ≥20 years (n=17,459) diagnosed with malignant tumors of the anterior mediastinum were identified from the Surveillance, Epidemiology, and End Results registry (SEER) and the Zentrum für Krebsregisterdaten (ZfKD) databases.

**Results:**

Among patients aged ≥20 years, TETs accounted for the most prevalent anterior mediastinal tumors (US/GER: 63%/64%), followed by lymphomas (14%/8%). For patients <20 years, predominant tumors included germ cell tumors (42%/14%), lymphomas (38%/53%), and TETs (10%/27%). The overall annual incidence of thymoma was 2.2/2.64 (US/GER) per million inhabitants and for thymic carcinomas 0.48/0.42. The male-to-female ratio was 1:1.09/1.03, and the mean age 59.48 ± 14.89/61.33 ± 13.94. Individuals with thymomas, but not thymic carcinomas, exhibited a 21%/29% significantly heightened risk of developing secondary malignancies compared to controls with non-thymic primary tumors.

**Discussion:**

This study provides a comparative analysis of anterior mediastinal tumors, particularly TETs, in the US and GER over the past two decades. Furthermore, it highlights a significantly elevated incidence of secondary malignancies in thymoma patients.

## 1 Background

The anterior mediastinum is the origin of a variety of tumors such as thymic epithelial tumors (TETs), lymphomas, mediastinal germ cell tumors, mesenchymal neoplasms, and metastases (1, 2). These tumors have also a specific age distribution with TETs being the most prominent group in adults and relatively rare in children (3).

Formally, TETs consist of thymomas, thymic carcinomas, and thymic neuroendocrine tumors (NET), however, in the following manuscript, we will use TET referring only to thymomas and thymic carcinomas. The recognition of thymomas and thymic carcinomas as separate entities has been introduced in the second edition of the WHO Classification of Tumors in 1999 and the nomenclature of the major thymoma types has been maintained ever since, although the field has dynamically evolved with both increased molecular pathology, radiology, and more interdisciplinary tumor board emphasis (2, 4–7). Based on the morphology and architecture of the epithelial cells and their proportion to lymphocytes, thymomas are divided into types A, AB, B1, B2, and B3 and other rare types. Thymic carcinomas are pathologically similar to extrathymic carcinomas and show typically a squamous differentiation (8, 9).

The biological behavior of TETs differs significantly between the entities. Whereas thymic carcinomas show a clear malignant potential with often locally advanced or metastatic disease at the time of diagnosis, type B thymomas grow slowly and tend to invade locally. Type A and AB thymomas metastasize rarely and were, therefore, envisioned as benign in the absence of invasive growth. Consequently, these cases were not reported to cancer registries. Since 2015, all thymomas and thymic carcinomas have been recognized as malignant tumors and subjected to reporting (10). Next to the standard TNM classification published by the Union for International Cancer Control (UICC), the Masaoka-Koga system is in use that allows significantly better discrimination for the early stages as compared to the UICC system (8). However, since it is not recorded in cancer registries, a national evaluation taking the Masaoka-Koga system into account is difficult to achieve.

Due to the rarity of TETs, the data collection and analysis are challenging and many of the current studies rely on single-center experiences making the epidemiological data incomplete and of limited comparability. Therefore, the aim of our study was a comprehensive evaluation of cancer registry data between the years 1999 and 2019 in two industrialized countries, the United States (US) and Germany (GER), focusing on a) tumors of the anterior mediastinum in general, b) TETs, c) secondary tumors in TET patients, d) comparison of the data between the two countries, and e) in the time course.

## 2 Methods

### 2.1 Descriptive statistics

The National Cancer Institute’s Surveillance, Epidemiology, and End Results (SEER) data provide information on cancer statistics among the United States population. These data were obtained from the SEER Research Limited-Field Data, 22 Registries, Nov 2021 Sub (2000-2019; SEER 22) (11) using SEER*Stat software (version 8.4.0.1). The German data were retrieved from the Centre for Cancer Registry Data (Zentrum für Krebsregisterdaten, ZfKD). The data were based on the epidemiological state cancer registry data [Version Epi2021_3 (12)], searching for the International Classification of Diseases for Oncology (ICD-O-3) morphology codes 8580 to 8589, topography codes C379 and C381, as well as other tumors from those patients. The analysis was performed using Microsoft Access (version 2202) and MS Excel (version 2202). To retrieve data on tumors of the anterior mediastinum, we searched the SEER 22 and ZfKD data for the ICD-O-3 topography codes C370, C379, and C381. The tumors were grouped by differentiation. Non-solid entities (e.g., leukemias) and unspecified diagnoses (e.g. neoplasm) were excluded. Descriptive statistics on relative frequencies, male-to-female ratios, age, incidence rates, and survival time were calculated as the mean ± standard deviation for continuous variables and frequencies or percentages for categorical variables. Figures were assembled using Corel PaintShop Pro (version 22.2.0.8).

### 2.2 Incidence rate

Incidence rates were calculated per year, per million inhabitants, and directly age-standardized to the 2000 US standard population from the Census P25-1130 or the population count of 2010 in the German states from the Federal Statistics Office (Destatis), respectively. For Germany, due to incomplete data sets and, following the recommendations of the ZfKD in some federal states, only the data for Schleswig-Holstein, Hamburg, Bremen, Rheinland-Pfalz, Saarland, Brandenburg, Mecklenburg-Vorpommern, Sachsen, and Thüringen were evaluated. Standard errors and 95% confidence intervals (CI) were calculated using the Tiwari modification for CIs (13). Cases were selected by the ICD-O-3 (8580–8586) or the “Site and Morphology Site recode ICD-O-3/WHO 2008 (for SIRs)” to split thymus from other endocrine organs. In the analysis, significantly fewer patients were diagnosed with specific thymoma subtypes in 1999 and 2000, since the exact subtyping of thymoma only became widely used with the second edition of the WHO Classification of Tumors in 1999. Therefore, for the calculation of incidence rates of thymoma subtypes, we excluded patients diagnosed in 1999-2000. In addition to the 22 registries SEER and ZfKD data for the time frame between 1999 or 2000 and 2019, we obtained the SEER Research Data, 8 Registries, Nov 2021 Sub (1975-2019; SEER 8) for the time between 1975 and 2019 (14).

To identify the time points in which the incidence rate trend significantly changed, we conducted a logistic regression analysis using the Joinpoint Regression Program (version 4.9.1.0) (15). The settings were as follows: dependent variable: age-adjusted rate with uncorrelated standard error; independent variable: annual year of diagnosis, cohorts defined by sex; log transformation. The regression model fits linear trends to the log-transformed incidence rates, with the inclination of the trend changing at the inflection points (joinpoints). The software analyzes trend data and fits the simplest model. For the test of whether more joinpoints are statistically significant compared to no joinpoints, we used the permutation test with a significance level of .01 and 9999 permutations. The number of data points determines the default maximum possible number of joinpoints. The slope of the model represents the annual percent change (APC). The average of the overall observed period is measured in the average annual percent change (AAPC). A two-sided statistical significance t-test on a 5% significance level is used to determine if the APC and AAPC differ significantly from zero.

### 2.3 Survival analysis

For survival analysis of the SEER and ZfKD data, the inclusion criteria for stratification by subtype were: only malignant tumors, only with histological confirmation, only cases with the ICD-O-3 8581 to 8586, only first malignancy, and no other diagnosed malignancies. To avoid as many potential sources of error as possible, such as other malignant tumors or misclassifications, we used restrictive inclusion criteria. The inclusion criteria for stratification by tumor stage were: only malignant tumors, only with histological confirmation, only with known TNM, only cases with the ICD-O-3 8581 to 8586, only first malignancy, no other diagnosed malignancies, only valid TNM (only 8 edition), exclusion of cases before the release of the 8^th^ edition TNM (December 2016), and exclusion of perioperative mortality defined as death within the first month of diagnosis. The exclusion of cases predating the release of the 8^th^ edition TNM is attributed to the absence of a widely adopted and uniform TNM classification system for thymus tumors until that time, leading to the utilization of numerous disparate systems or the lack thereof (16). The tumor stages were classified using the Union for International Cancer Control (UICC) criteria of the 8^th^ edition. The analyzes were performed using SPSS (version 27.0.1.0). The log-rank test was performed to compare the estimated survival between the cohorts.

### 2.4 Consecutive malignancies

For the evaluation of consecutive malignancies, we identified both German and United States patients with TETs. The crude rate of the different consecutive malignancies of those patients between 2000 and 2019 was calculated. We determined the ratio between the rate of consecutive tumors and the respective number of primary tumors. As a control group, we chose all primary cancer patients in the SEER 22 cohort excluding epithelial tumors of the thymus. The rate of subsequent malignancies was reported as a ratio to the control group. We conducted a two-tailed Fisher`s exact test. Due to the comprehensive volume of data, the consecutive tumors were grouped according to lineage differentiation (i.e., for instance, lymphomas as hematopoietic neoplasms and both adenocarcinomas and squamous cell carcinomas as epithelial and so on). To avoid assessing erroneous multiple reports to the cancer registry, we excluded repeated reports of thymoma and thymic carcinoma cases. As no primary basal cell and squamous carcinomas of the skin are reported to the SEER registry, those tumors were excluded from the German cohort as well. Moreover, we calculated second tumors without considering other subsequent tumors.

## 3 Results

### 3.1 Anterior mediastinal tumors in the United States and Germany

**Figure 1:**
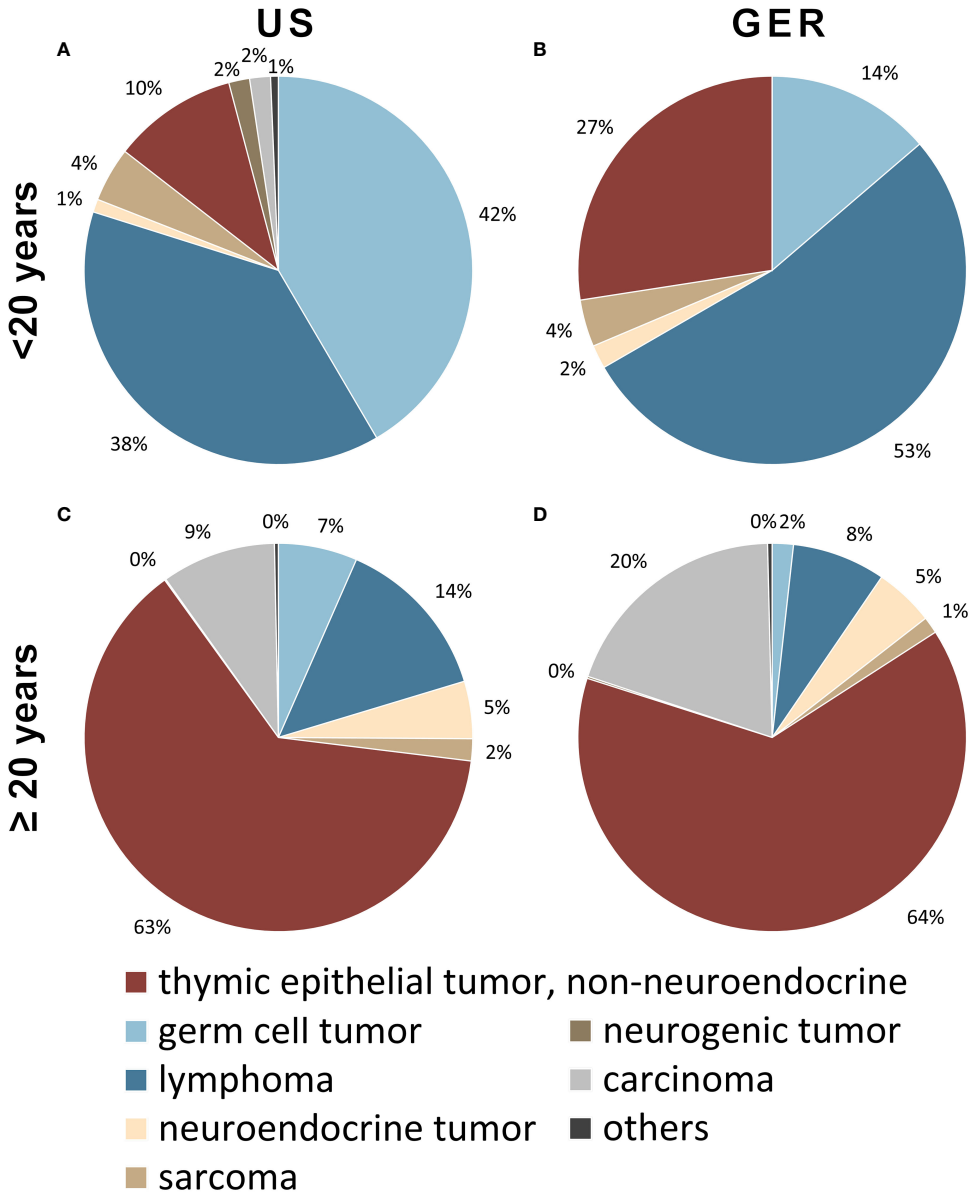
Tumors of patients younger than 20 years of the anterior mediastinum including thymus in the **(A)** United States (US; n=427) and **(B)** German (GER; n=51) cohort. Tumors of 20-year-old patients or older in the **(C)** US (n=12736) as well as **(D)** Germany (n=4723). In younger patients, most tumors are lymphomas and germ cell tumors, while in older patients the majority is comprised of non-neuroendocrine thymic epithelial tumors. Cases of nondescript carcinomas were considered a separate category (grey).

Data on anterior mediastinal tumors obtained from the US (2000–2019) and GER (1999–2019) cancer registries were analyzed for the distribution of tumor entities in patients below 20 years of age and older (**Figure 1**). In patients younger than 20 years, the most frequent entities were: germ cell tumors (US: 42% and Germany: 14%), lymphomas (38% and 53%), and TETs (10% and 27%). This ratio changed in patients aged 20 years or more with TETs representing the by far most frequent entity (63% and 64%) followed by lymphomas (14%, 8%), carcinoma not otherwise specified (9%, 20%), germ cell tumors (7%, 2%), neuroendocrine tumors (5%, 5%) and sarcomas (2% and 1%). The notable disparities observed between the percentages of German and United States numbers can be attributed primarily to the relatively low number of overall patients in the German cohort of young patients, consisting of only 51 individuals, in contrast to the substantial sample size of 427 patients in the US cohort.

In the US, the inquiry identified 8,171 patients diagnosed with a TET with a male-to-female ratio of 1.09, the mean age of primary diagnosis 59.48 years (SD, 14.89), and the incidence rate of 2.68 per million inhabitants per year at risk. In Germany, 3,081 TET patients were found with a male-to-female ratio of 1.03, a mean age of 61.33 years (± 13.94), and an overall incidence rate of 3.06 per million inhabitants per year. The exact tumor subtype was reported in 5,979 (73.17%) and 2,905 cases (94.29%) in the United States and German data sets, respectively (**Table 1**). The differences between both countries regarding the frequency, male-to-female ratio, age, mean survival, and incidence rate were statistically not significant, except for the mean survival of thymic carcinoma patients, which was significantly better in Germany (**Table 1**).

**Table 1:** Comparison of epidemiological features of thymomas and thymic carcinoma in Germany (GER) in 1999-2019 and the United States (US) in 2000-2019.

Entity	Relative frequency [%]		Male-to-female ratio		Age [mean ± SE]		Mean Survival [months ± SE]		Mean incidence rate [per million]	
	US	GER	US	GER	US	GER	US	GER	US	GER
**A thymoma**	9.9	11.2	1:1.13	1:1.04	67.0 ± 12.6	68.5 ± 11.0	139.4 ± 8.3	137.6 ± 9.1	0.20	0.25
**AB thymoma**	19.2	25.8	1:0.95	1:0.91	61.1 ± 12.9	63.8 ± 11.8	160.0 ± 5.6	176.5 ± 8.0	0.39	0.57
**B1 thymoma**	13.5	13.9	1:0.82	1:0.81	56.3 ± 14.8	58.6 ± 15.2	153.4 ± 5.9	162.3 ± 10.2	0.27	0.30
**B2 thymoma**	16.1	19.2	1:0.95	1:0.91	55.6 ± 15.2	57.9 ± 14.5	157.5 ± 6.1	145.9 ± 7.9	0.33	0.42
**B3 thymoma**	17.5	14.3	1:1.07	1:0.98	58.3 ± 14.9	61.4 ± 13.1	146.0 ± 5.4	130.9 ± 7.1	0.36	0.31
**Thymic carcinoma**	23.9	15.6	1:1.61	1:1.32	61.0 ± 14.0	60.4 ± 14.6	72.5 ± 3.7	98.1 ± 5.6	0.48	0.42

**Figure 2:**
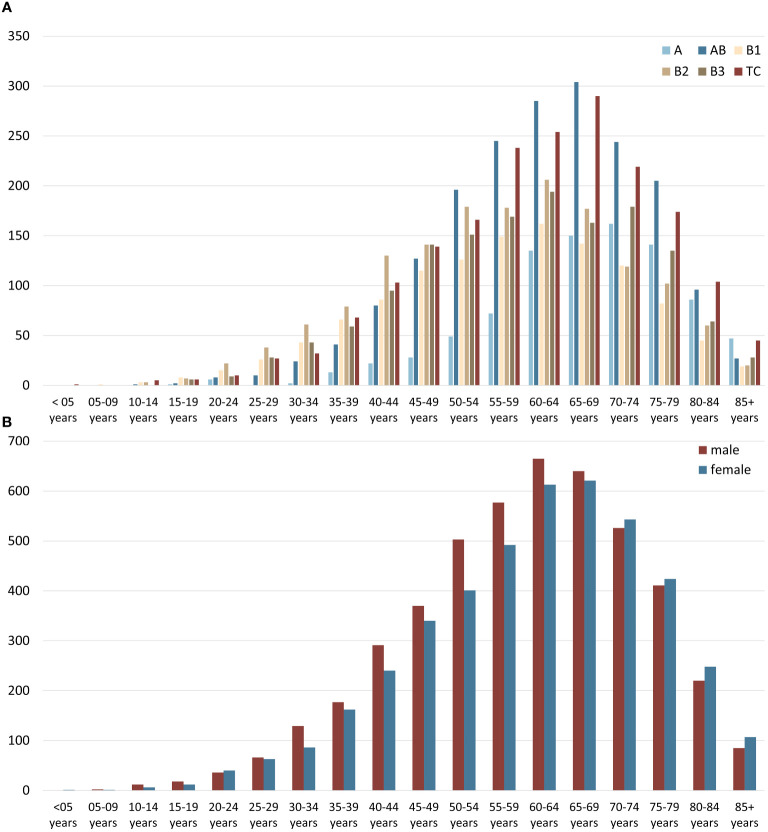
**(A)** Age distribution of thymic epithelial tumors, non-neuroendocrine (TETs) subtypes, and **(B)** age and sex distribution of all TETs taking the US (2000–2019) and German (1999–2019) data together.

Most epithelial tumors of the thymus were diagnosed between 40 and 60 years of age, overall, with only minor variations between the sexes. The distribution of subtypes concerning the age at diagnosis also showed a consistent pattern (**Figure 2**). However, when conducting a subgroup analysis, we observed notable differences between the United States and Germany. Thymic carcinomas were diagnosed relatively frequently in the United States, while in Germany, AB thymomas were reported more frequently (**Supplementary Figure 1**). Additionally, in a comprehensive cohort analysis of patients with malignancies of the anterior mediastinum spanning from 1975 to 2019, it can be found that the overall incidence rate of Thymus tumors increased over time, primarily driven by the rise in carcinomas (**Supplementary Figure 2**).

### 3.2 Incidence analysis of thymic epithelial tumors subtypes

**Figure 3:**
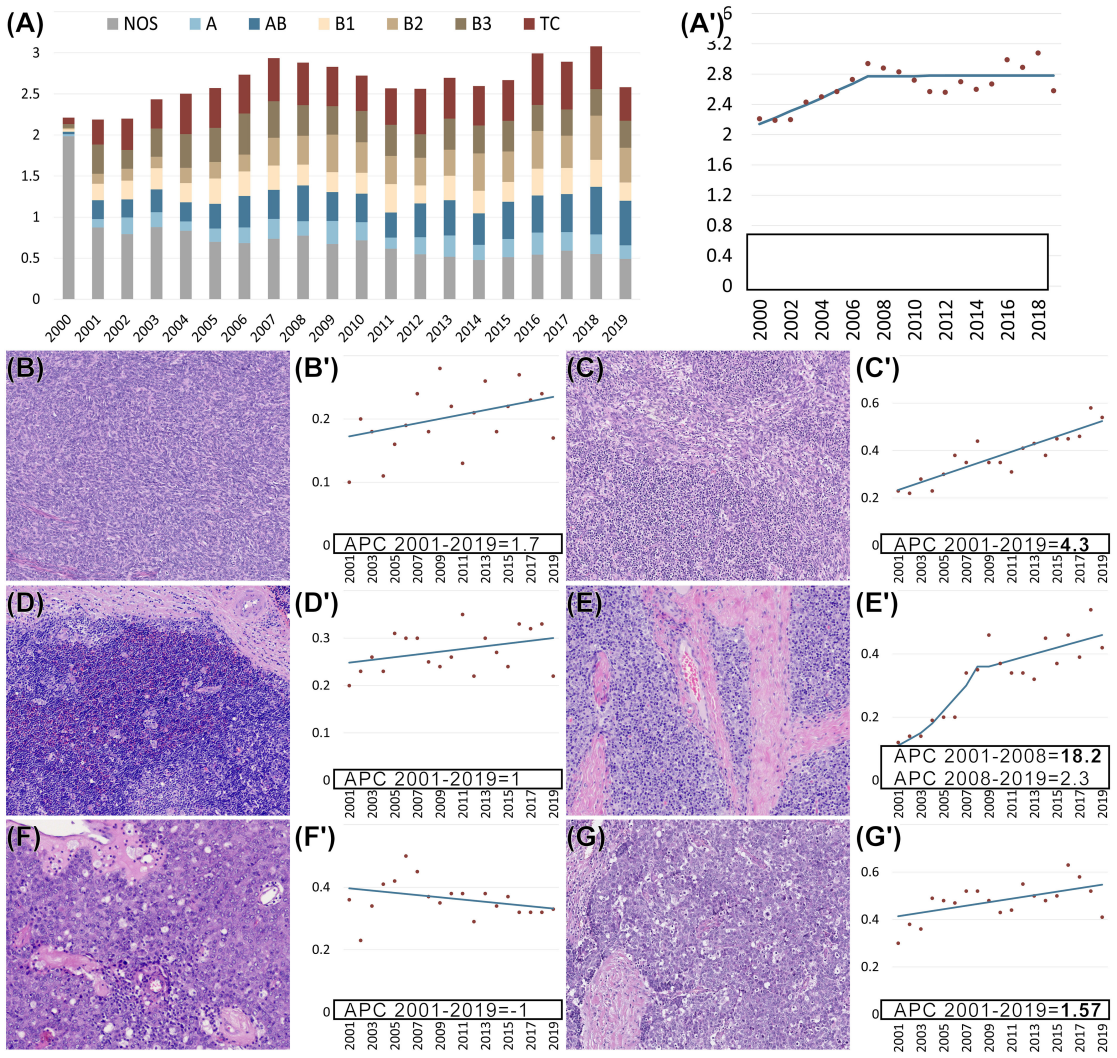
**(A, A’–G’)** Age-adjusted incidence rates per million (y-axis) of thymomas not otherwise specified (NOS) and specific subtypes as well as thymic carcinomas (TCs) in the US from 2000-2019 (x-axis). Regression analysis of age-adjusted incidence rates per million for all non-neuroendocrine epithelial tumors of the thymus **(A’)** and each subtype separately **(B’–G’)**. Morphology of different subtypes of epithelial tumors of the thymus: **(B)** A thymoma, **(C)** AB thymoma, **(D)** B1 thymoma, **(E)** B2 thymoma, **(F)** B3 thymoma, and **(G)** thymic carcinoma. **(B’–G’)** Separate regression analysis of age-adjusted incidence rates per million for each of the subtypes. **(A’–G’)** Bold font indicates that the annual percent change (APC) is significantly different from zero at the p =.05 level. **(B–G)** Hematoxylin and eosin (HE) staining. **(B–E, G)** 100x magnification. **(F)** 200x magnification.

To reveal changes in the incidence of TETs during the observation period, an incidence analysis was performed. This analysis shows incidence rate trends and identifies joinpoints, at which the inclination of the trend changes. In the regression model for the evaluation of the age-adjusted incidence rates of TETs in the US, one such joinpoint was identified in 2007 (**Figure 3**). Overall, the incidence rates increased until 2007 and remained stable thereafter at 2.8 per million inhabitants per year. For both sexes, the average annual percent change (AAPC) was statistically significant on a 5% significance level [1.4%, 95% CI (0.3; 2.5), *p* =.01]. For female patients, the AAPC was 1.5% [95% CI (0.7; 2.3), *p* <.01]. For male patients the AAPC was lower with 0.6% [95% CI (-0.1; 1.3), *p*=.07]. Regarding tumor subtypes, AB thymomas showed a rise in incidence with an AAPC of 4.3% [95% CI (3.2; 5.5), *p* <.01]. This change occurred for both sexes (male 4.9%, female 3.9%, both *p*< .01). B2 thymomas showed an overall AAPC of 8.2% [95% CI (4.5; 11.9); *p* <.01]. This thymoma subtype increased more for females (AAPC 8.7% [95% CI (4.1; 13.5); *p* <.01] than for males (AAPC 5.9% [95% CI (3.6; 8.2); *p* <.01]. The incidence of thymic carcinomas also increased in the observed period (AAPC 1.6% [95% CI (0.3; 2.9); *p* =.02]. However, there were no sex differences, both showing an AAPC of 1.5%.

### 3.3 Survival analysis and UICC stage of TETs

**Figure 4:**
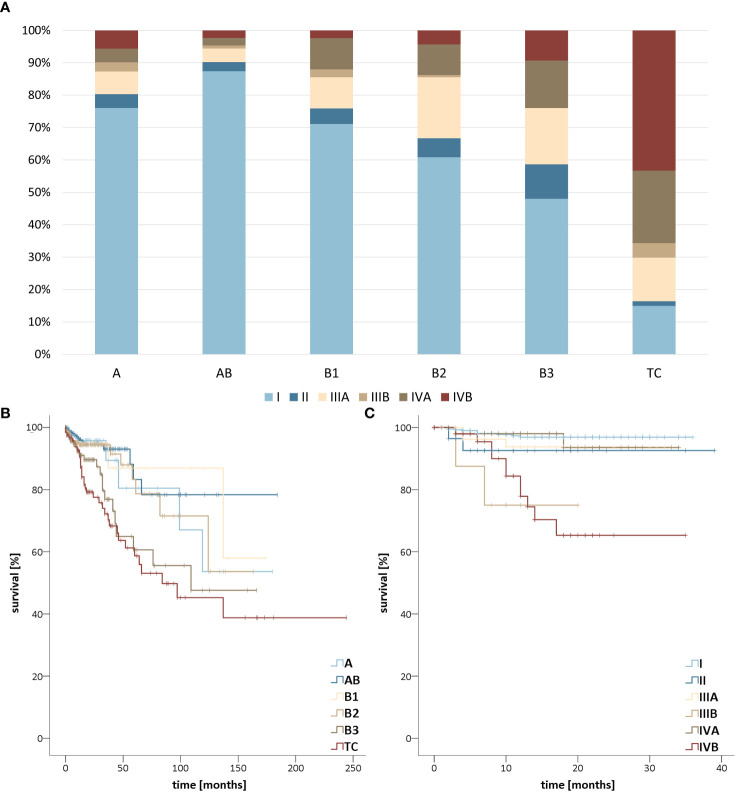
**(A)** Stage distribution of thymomas and thymic carcinomas in the United States (US) from 2018-2019 and Germany (GER) from 2017-2019 according to the Union for International Cancer Control (UICC) classification. Survival stratified **(B)** by TETs subtype in the US from 2000-2019 as well as GER from 1999-2019 and **(C)** by Union for International Cancer Control (UICC) stage of thymomas and thymic carcinomas in the US from 2018-2019 and GER from 2017-2019.

For survival analyzes, 1,869 (GER) and 4,156 (US) patients met the inclusion criteria for analysis by subtype and a total of 648 patients met the inclusion criteria for analysis by UICC stage. Regarding the UICC stages, AB thymoma most frequently showed low tumor stages followed by A thymomas to B1, B2, and B3 thymomas, whereas thymic carcinoma already reached stage IVB in more than 40% of cases and was detected in the first two stages in only about 15% (**Figure 4**). This was also reflected in the Kaplan-Meier curves, which differ significantly (*p <*.01). Surprisingly, UICC stage IIIB showed a significantly worse outcome than UICC stage IVA (*p* =.01). In the comparison between the United States and Germany, only thymic carcinoma patients diagnosed in GER show a significantly better prognosis (**Supplementary Figure 3**, *p* =.01). For the mean survival values, see **Table 1**.

### 3.4 Occurrence of secondary malignancies in TET patients

In general, tumor patients show an increased incidence of secondary malignancies. We aimed to test the hypothesis that TET patients suffer from a disproportionally higher incidence of secondary tumors as compared to non-TET tumor patients (17). To this end, we compared the incidence of secondary malignant tumors in our thymoma and thymic carcinoma cohorts with a control group comprising 11,748,248 patients, consisting of all other primary cancer patients in the SEER 22 cohort excluding TET patients.

For the evaluation of sequential tumors, we identified 3806 and 2829 primary thymoma and 1166 and 376 primary thymic carcinoma patients in the US and German cohorts, respectively. In the thymoma cohort, 11.53% (US) and 13.18% (GER) suffered from a secondary malignancy.

Most secondary tumors in the US cohort were lung cancers (n=97, of which 70 were of epithelial origin, e.g., adenocarcinomas or squamous carcinomas), followed by adenocarcinomas of the prostate (n= 64), and thyroid carcinomas (n= 16). The most frequent subsequent tumors of thymoma patients in the GER cohort were lung cancers (n= 45, including 35 cases of epithelial differentiation), prostate cancer (n= 25), and breast -adeno-carcinomas (n= 7). In the thymic carcinoma cohort, secondary tumors emerged in 8.75% (US) and 10.90% (GER) patients, respectively. In the control group comprising 11,748,248 patients, 9.53% of patients had secondary cancers. As compared to the non-TET cohort, patients with thymomas were at a significantly higher risk of developing a secondary neoplasm in the US and Germany (**Table 2**). In contrast, in patients with thymic carcinoma, the risk of a secondary tumor did not differ from the non-TET cohort.

**Table 2:** The relative risk of patients with thymomas and thymic carcinoma to develop consecutive malignancies in the United States (2000–2019) and Germany (1999-2019).

Second malignancy	Thymoma [relative risk]				Thymic carcinoma [relative risk]			
	US	*p*	GER	*p*	US	*p*	GER	*p*
**Hematopoietic**	1.27	.06	**1.46**	<.01	1.41	.12	1.04	.82
**Epithelial**	**1.18**	<.01	**1.25**	<.01	0.80	.07	0.76	.25
**Mesenchymal**	**2.04**	.01	**2.01**	.03	1.77	.30	1.37	.52
**Neuroectodermal**	1.13	.39	1.06	.71	1.0	1	1.45	.12
**Overall**	**1.21**	<.01	**1.29**	<.01	0.97	.74	1.02	.74

Bold font indicates a statistically significant difference (*p* <.05).

## 4 Discussion

The epidemiology of epithelial tumors of the thymus is not comprehensively documented due to their rarity and the fact that the current categorization was only introduced in 1999. Regarding survival data, the situation is even more difficult to assess, since only the latest edition of the UICC classification has introduced a uniform system for thymomas. In addition, previously published unicentric studies usually did not come up with sufficient case numbers to stratify survival data feasibly. This is complicated by the fact that neither the German nor the United States cancer registries collect data on Masaoka-Koga stages.

Our data show that in adults, the distribution of anterior mediastinal tumors did not show large differences between US and GER, with TETs representing the by far most frequent entity in this age group (63% and 64% in the US and Germany, respectively). In patients younger than twenty years, we observed germ cell tumors and lymphomas in 38%/53% (US/GER) and 42%/14%, respectively, whereas TETs accounted for only 10% (US) and 27% (GER) of tumors. As stated before, the notable disparities observed between the percentages of German and United States numbers can be attributed primarily to the relatively low number of overall patients in the German cohort. The relatively lower incidence of neurogenic tumors in our cohorts, compared to other studies, can be attributed to our exclusive focus on the anterior mediastinum, where such tumors are less prevalent (3, 18–20).

In our cohort, the overall incidence of TETs was 2.68 (US) and 3.06 (GER) per million inhabitants per year with only slight differences between the two countries in terms of subtypes and gender and age distribution. This is the first nationwide incidence study for Germany. For the US, the incidence is higher than the previously published data of 1.3 per million person-years considering thymomas between 1973 and 2006 (9). However, these incidence rates are limited by the fact that thymic carcinomas might be inconsistently considered, especially considering that it was only established as a separate entity in 2004 (21) and the fact that non-invasive thymomas were envisioned as benign tumors and excluded from reporting. This has gradually changed based on several publications to be finally defined in the fourth edition of the WHO Classification of Tumors of the Thymus so since then all thymomas have been classified as malignant (10).

Previous reports in the fifth edition of the WHO classification have suggested a higher occurrence of type A, AB, and B1 thymomas in females. However, our analysis of both the United States and the German cohort revealed that among the different thymoma subtypes, the incidences of AB, B1, and B2 thymomas were slightly higher in men, while the occurrence of A and B3 thymomas showed no significant difference between genders. These findings are mostly in line with the findings reported in several previous studies (22–25). The age distribution conforms closely to a normal distribution with sixty years as the mean. Only minor deviations were found in the subtype analysis. For example, type B1 and B2 thymomas were more frequent in younger patients than in older ones, while A thymomas and thymic carcinomas showed a tendency to occur more frequently in older patients. The incidence rates of TETs increased during the observed period. This development is certainly due to a certain extent to both improved radiological diagnostics and increased awareness of this tumor group (26). Considering the changes in incidence rates over the observed period, increasing trends were particularly evident for AB and B2 thymomas, with B2 thymomas particularly markedly increasing up to 2008.

Some reservations regarding the diagnosis of thymic carcinomas in cancer registry data need to be considered. First, there are many nondescript carcinomas in the anterior mediastinum classified, accounting for 9% (US) and 20% (GER) of all tumors reported. To a large extent, these tumors are most likely misclassified, since it can be assumed that no other epithelial tumors besides carcinomas of the thymus occur at this localization. The closest localization where carcinomas would be expected is the skin and lung, each of which has its ICD-O-3 code. Overall, the exact number of thymic carcinomas cannot be determined, although a higher number than reported here can be assumed. Consequently, the percentage of epithelial tumors of the thymus in adult patients could be as high as 72% to 84%. Second, these differences could preferentially affect a certain subgroup of patients, for example, patients with a higher TNM stage or lower differentiation. Consequently, the different survival times between the United States and German cohorts may be distorted. Despite these uncertainties, an unambiguous view of thymic carcinoma can be derived from the similarity of results for incidence and median survival between the data from Germany and the United States.

An increased incidence of secondary tumors has been reported in thymoma patients, often attributed to altered T-cell immune function or genetic predisposition (9). Nevertheless, a clear consensus regarding the specific tumor types that are more prevalent in thymoma patients remains elusive. Discrepancies in previous research findings have contributed to the need for further investigation to establish a better understanding of this association. For example, studies have described an association with hematological disorders (27), thyroid carcinomas (28), and gastrointestinal tract tumors (17, 24, 29), with the rate of increased occurrences varying significantly. In addition, the control groups in the aforementioned studies were of questionable size and comparability. Given the low number of thymoma patients, the subgroup of individuals with secondary tumors is inherently small. As a result, it is readily apparent that the subset of patients exhibiting secondary tumors is intrinsically diminutive, rendering statistical analysis challenging. To account for this circumstance, in our study, we grouped tumors by lineage differentiation, because it can be presumed that entities with similar lineages share comparable biological features. This approach mitigates the risk of statistical overvaluation resulting from individual rare secondary tumors by preventing them from causing undue influence due to their absence in the control group. The incidence of secondary malignancies in patients with thymomas and thymic carcinomas was compared with a control group of a large number of non-TET cancer patients in the same period. In both the German and US cohorts, thymomas predisposed significantly more for malignancies with epithelial, hematopoietic, and mesenchymal lineage differentiation. Neuroectodermal tumors did not occur more frequently in thymoma patients. These findings using large and matched control groups support previous studies indicating that thymoma patients are at a significantly higher risk of developing secondary malignancies compared to other tumor patients (17). In contrast, the incidence of secondary malignancies in patients with thymic carcinomas did not differ from the ones with non-TET primary tumors. These results allude to the hypothesis that the thymopoiesis typically occurring in thymomas but not in thymic carcinomas might be prone to generate dysfunctional T cells and thereby the decisive reason for a defective self-tolerance. This theory is supported, among other things, by the higher incidence of autoimmune diseases, as well as the increased likelihood in this patient group to develop immune-related adverse events during immune checkpoint blockade with Pembrolizumab (30). However, further research is needed to better understand the underlying mechanisms. In addition, these findings highlight the importance of closer monitoring of thymoma patients to detect and manage subsequent malignancies.

The present study has several limitations. First, it was a retrospective analysis of cancer registry data. This led to a particular limitation of available information on the respective patients, so that, for example, Masaoka-Koga stages were missing. In addition, the data are susceptible to misclassification mistakes due to a possibly limited experience of some physicians dealing with rare entities. Conversely, however, single-center studies are subject to even more significant limitations due to the rarity of the tumors and are increasingly influenced by individual investigators. Concerning the relative frequency of secondary tumors, an additional limitation is that only a control group from the United States but not from Germany was available. However, due to the comparable medical and demographic state of development of both countries, only minor deviations can be assumed. Concerning the elevated rate of secondary tumors in thymoma patients, it is essential to note certain limitations as well. The restricted overall tumor count poses challenges in developing a detailed subclassification based on therapy for statistical analysis. The precise nature of chemotherapy, immunotherapies (such as PD-L1 immune checkpoint inhibition), the dosage of radiotherapy, and the respective time intervals leading to the development of subsequent tumors must be considered to ascertain potential causality. In both thymoma and thymic cancers, curative intent resection remains the preferred treatment for resectable disease. Additionally, patients may undergo radiation with or without chemotherapy, contingent upon the Masaoka stage. In the case of stage II tumors, radiation may be contemplated for high-risk cases, while for stage III and IV tumors, radiation is usually part of a combination therapy (31, 32). For our cohorts, this implies that, due to the advanced stage of the tumor, a higher proportion of patients with thymic carcinoma likely underwent combination therapy involving radiation compared to patients with thymoma. Considering the carcinogenic effects of radiation, an augmented incidence of secondary tumors could reasonably be anticipated. Consequently, even though the survival rate of thymic carcinoma patients is significantly diminished, the inclination would be to expect a heightened frequency of secondary tumors in thymic carcinoma patients. However, the observed data does not support this expectation, thereby reinforcing the conclusion that the increased rate of secondary tumors in thymic patients is attributable to factors other than therapy.

In summary, our results demonstrate the age-specific tumor distribution of the tumors in the anterior mediastinum in a nationwide manner in the US and Germany over a time range of 20 years. The study identified stable incidence rates of TETs over time and revealed significant differences in survival outcomes among tumor subtypes and stages. Additionally, it highlighted an increased risk of secondary malignancies in thymoma patients compared to patients with non-TET primaries. These results contribute to our understanding of anterior mediastinal tumors, emphasizing the importance of tailored approaches based on tumor subtypes and the need for monitoring secondary malignancies in TET patients.

## Data availability statement

The data analyzed in this study is subject to the following licenses/restrictions: The data that support the findings of this study are available from the Centre for Cancer Registry Data (Zentrum für Krebsregisterdaten, ZfKD) and Surveillance, Epidemiology, and End Results (SEER) Program (www.seer.cancer.gov). Restrictions apply to the availability of these data, which were used under license for this study. Requests to access these datasets should be directed to www.seer.cancer.gov and https://www.krebsdaten.de/Krebs/DE/Home/homepagenode.html.

## Ethics statement

Ethical approval was not required for the study involving humans in accordance with the local legislation and institutional requirements. Written informed consent to participate in this study was not required from the participants or the participants’ legal guardians/next of kin in accordance with the national legislation and the institutional requirements.

## Author contributions

TG: Conceptualization, Data curation, Formal analysis, Investigation, Methodology, Resources, Software, Validation, Visualization, Writing – original draft, Writing – review & editing. SS: Data curation, Methodology, Writing – review & editing. AM: Conceptualization, Validation, Writing – review & editing. WR: Supervision, Writing – review & editing. SP: Conceptualization, Data curation, Formal analysis, Validation, Writing – original draft, Writing – review & editing.

## Supplementary material

Supplementary Figure 1
**(A)** Age distribution of thymomas and thymic carcinomas in the **(A)** US from 2000-2019 and **(B)** Germany from 1999-2019. **(B)** Age and sex distribution of all epithelial tumors of the thymus in the **(C)** US from 2000-2019 and **(D)** Germany from 1999-2019.

Supplementary Figure 2 Regression analysis of age-adjusted incidence rates per million between 1975 and 2019 of **(A)** all tumors of the thymus regardless of histology and **(B)** of all tumors of the thymus excluding epithelial tumors of the thymus, which are mostly comprised of tumors classified as squamous cell carcinomas (n=746), adenocarcinomas (n=502), and unspecified neoplasms (n=103).

Supplementary Figure 3 Stage distribution of thymomas and thymic carcinomas in the **(A)** US from 2018-2019 and **(B)** Germany from 2017-2019. Survival stratified by subtype in the **(C)** US from 2000-2019 and **(D)** Germany from 1999-2019. **(E)** Significantly different survival of thymic carcinomas between the US and Germany.

## References

[1] Roden AC Fang W Shen Y Carter BW White DB Jenkins SM. Distribution of mediastinal lesions across multi-institutional, international, radiology databases. J Thorac Oncol (2020) 15(4):568–79. doi: 10.1016/j.jtho.2019.12.108 31870881

[2] Marx A Chan JKC Chalabreysse L Dacic S Detterbeck F French CA. The 2021 WHO classification of tumors of the thymus and mediastinum: what is new in thymic epithelial, germ cell, and mesenchymal tumors? J Thorac Oncol (2022) 17(2):200–13. doi: 10.1016/j.jtho.2021.10.010 34695605

[3] Stachowicz-Stencel T Orbach D Brecht I Schneider D Bien E Synakiewicz A. Thymoma and thymic carcinoma in children and adolescents: a report from the European Cooperative Study Group for Pediatric Rare Tumors (EXPeRT). Eur J Cancer (2015) 51(16):2444–52. doi: 10.1016/j.ejca.2015.06.121 26259494

[4] Marx A Belharazem D Lee DH Popovic ZV Reißfelder C Schalke B. Molecular pathology of thymomas: implications for diagnosis and therapy. Virchows Arch (2021) 478(1):101–10. doi: 10.1007/s00428-021-03068-8 PMC7966134 33674910

[5] Radovich M Pickering CR Felau I Ha G Zhang H Jo H. The integrated genomic landscape of thymic epithelial tumors. Cancer Cell (2018) 33(2):244–58.e10. doi: 10.1016/j.ccell.2018.01.003 29438696 PMC5994906

[6] Levine GD Rosai J. Thymic hyperplasia and neoplasia: a review of current concepts. Hum Pathol (1978) 9(5):495–515. doi: 10.1016/S0046-8177(78)80131-2 361541

[7] Board W. Thoracic tumours: WHO classification of tumours. Lyon, France: International Agency for Research on Cancer. (2021).

[8] Oramas DM Moran CA. Thymoma: Histologically a heterogenous group of tumors. Semin Diagn Pathol (2022) 39(2):99–104. doi: 10.1053/j.semdp.2021.06.002 34147302

[9] Engels EA. Epidemiology of thymoma and associated Malignancies. J Thorac Oncol (2010) 5:S260–5. doi: 10.1097/JTO.0b013e3181f1f62d PMC2951303 20859116

[10] Marx A Chan JK Coindre JM Detterbeck F Girard N Harris NL. The 2015 world health organization classification of tumors of the thymus: continuity and changes. J Thorac Oncol (2015) 10(10):1383–95. doi: 10.1097/JTO.0000000000000654 PMC4581965 26295375

[11] Institute NC. Surveillance, Epidemiology, and End Results (SEER) Program (www.seer.cancer.gov) SEER*Stat Database: Incidence - SEER Research Limited-Field Data, 22 Registries, Nov 2021 Sub (2000-2019) - Linked To County Attributes - Time Dependent (1990-2019) Income/Rurality, 1969-2020 Counties, National Cancer Institute, DCCPS, Surveillance Research Program, released April 2022, based on the November 2021 submission. (2022).

[12] Zentrum für Krebsregisterdaten im Robert K-I. Datensatz des ZfKD auf Basis der epidemiologischen Landeskrebsregisterdaten Epi2021_3, verfügbare Diagnosejahre bis 2019. ZfKD - German Center Cancer Registry Data at RKI (2023).

[13] Tiwari RC Clegg LX Zou Z. Efficient interval estimation for age-adjusted cancer rates. Stat Methods Med Res (2006) 15(6):547–69. doi: 10.1177/0962280206070621 17260923

[14] Institute NC Surveillance, Epidemiology, and End Results (SEER) Program. (www.seer.cancer.gov) SEER*Stat Database: Incidence - SEER Research Data, 8 Registries, Nov 2021 Sub (1975-2019) - Linked To County Attributes - Time Dependent (1990-2019) Income/Rurality, 1969-2020 Counties, National Cancer Institute, DCCPS, Surveillance Research Program, released April 2022, based on the November 2021 submission. Surveillance Systems Branch, Surveillance Research Program, Division of Cancer Control and Population Sciences: National Cancer Institute (2022).

[15] Kim HJ Fay MP Feuer EJ Midthune DN. Permutation tests for joinpoint regression with applications to cancer rates. Stat Med (2000) 19(3):335–51. doi: 10.1002/(SICI)1097-0258(20000215)19:3<335::AID-SIM336>3.0.CO;2-Z 10649300

[16] Ahmad U. The eighth edition TNM stage classification for thymic tumors: What do I need to know? J Thorac Cardiovasc Surg (2021) 161(4):1524–9. doi: 10.1016/j.jtcvs.2020.10.131 33468329

[17] Pan C-C Chen PC-H Wang L-S Chi K-H Chiang H. Thymoma is associated with an increased risk of second Malignancy. Cancer (2001) 92(9):2406–11. doi: 10.1002/1097-0142(20011101)92:9<2406::AID-CNCR1589>3.0.CO;2-7 11745297

[18] Rothstein DH Voss SD Isakoff M Puder M. Thymoma in a child: case report and review of the literature. Pediatr Surg Int (2005) 21(7):548–51. doi: 10.1007/s00383-005-1419-4 15926048

[19] Rod J Orbach D Verité C Coze C Stephan JL Varlet F. Surgical management of thymic epithelial tumors in children: lessons from the French Society of Pediatric Oncology and review of the literature. Pediatr Blood Cancer (2014) 61(11):1910–5. doi: 10.1002/pbc.25159 25130986

[20] Williamson SR Ulbright TM. Germ cell tumors of the mediastinum. In A. M. Marchevsky, M. R. Wick (Eds.). Pathology of the mediastinum. pp. 146–68. New York: Cambridge University Press. (2014).

[21] Ströbel P Marx A Zettl A Müller-Hermelink HK. Thymoma and thymic carcinoma: an update of the WHO classification 2004. Surg Today (2005) 35(10):805–11. doi: 10.1007/s00595-005-3047-y 16175459

[22] Engels EA Pfeiffer RM. Malignant thymoma in the United States: demographic patterns in incidence and associations with subsequent Malignancies. Int J Cancer (2003) 105(4):546–51. doi: 10.1002/ijc.11099 12712448

[23] Thomas CR Wright CD Loehrer PJ. Thymoma: state of the art. J Clin Oncol (1999) 17(7):2280–9. doi: 10.1200/JCO.1999.17.7.2280 10561285

[24] Wilkins KB Sheikh E Green R Patel M George S Takano M. Clinical and pathologic predictors of survival in patients with thymoma. Ann Surg (1999) 230(4):562–72. doi: 10.1097/00000658-199910000-00012 PMC1420905 10522726

[25] Couture MM Mountain CF. Thymoma. Semin Surg Oncol (1990) 6(2):110–4. doi: 10.1002/ssu.2980060209 2315600

[26] Roden AC Ahmad U Cardillo G Girard N Jain D Marom EM. Thymic carcinomas-A concise multidisciplinary update on recent developments from the thymic carcinoma working group of the international thymic Malignancy interest group. J Thorac Oncol (2022) 17(5):637–50. doi: 10.1016/j.jtho.2022.01.021 PMC11080660 35227908

[27] Souadjian JV Silverstein MN Titus JL. Thymoma and cancer. Cancer. (1968) 22(6):1221–5. doi: 10.1002/1097-0142(196811)22:6<1221::AID-CNCR2820220619>3.0.CO;2-7 5705783

[28] LeGolvan DP Abell MR. Thymomas. Cancer. (1977) 39(5):2142–57. doi: 10.1002/1097-0142(197705)39:5<2142::AID-CNCR2820390531>3.0.CO;2-Q 558046

[29] Gray GF Gutowski WT. Thymoma. A clinicopathologic study of 54 cases. Am J Surg Pathol (1979) 3(3):235–49. doi: 10.1097/00000478-197906000-00006 532853

[30] Lippner EA Lewis DB Robinson WH Katsumoto TR. Paraneoplastic and therapy-related immune complications in thymic Malignancies. Curr Treat Options Oncol (2019) 20(7):62. doi: 10.1007/s11864-019-0661-2 31227926

[31] Falkson CB Bezjak A Darling G Gregg R Malthaner R Maziak DE. The management of thymoma: A systematic review and practice guideline. J Thorac Oncol (2009) 4(7):911–9. doi: 10.1097/JTO.0b013e3181a4b8e0 19557895

[32] Liu J Govindarajan A Williams TM Kim J Erhunmwunsee L Raz D. An updated review on radiation treatment management in thymus cancers. Clin Lung Cancer (2022) 23(7):561–70. doi: 10.1016/j.cllc.2022.07.004 35941046

