# Supplementary figures and images for: Epidemiology of thymomas and thymic carcinomas in the United States and Germany, 1999-2019
Source: Front Oncol. 2024 Jan 9;13:1308989. doi: 10.3389/fonc.2023.1308989 (PMC10805269; doi:10.3389/fonc.2023.1308989)

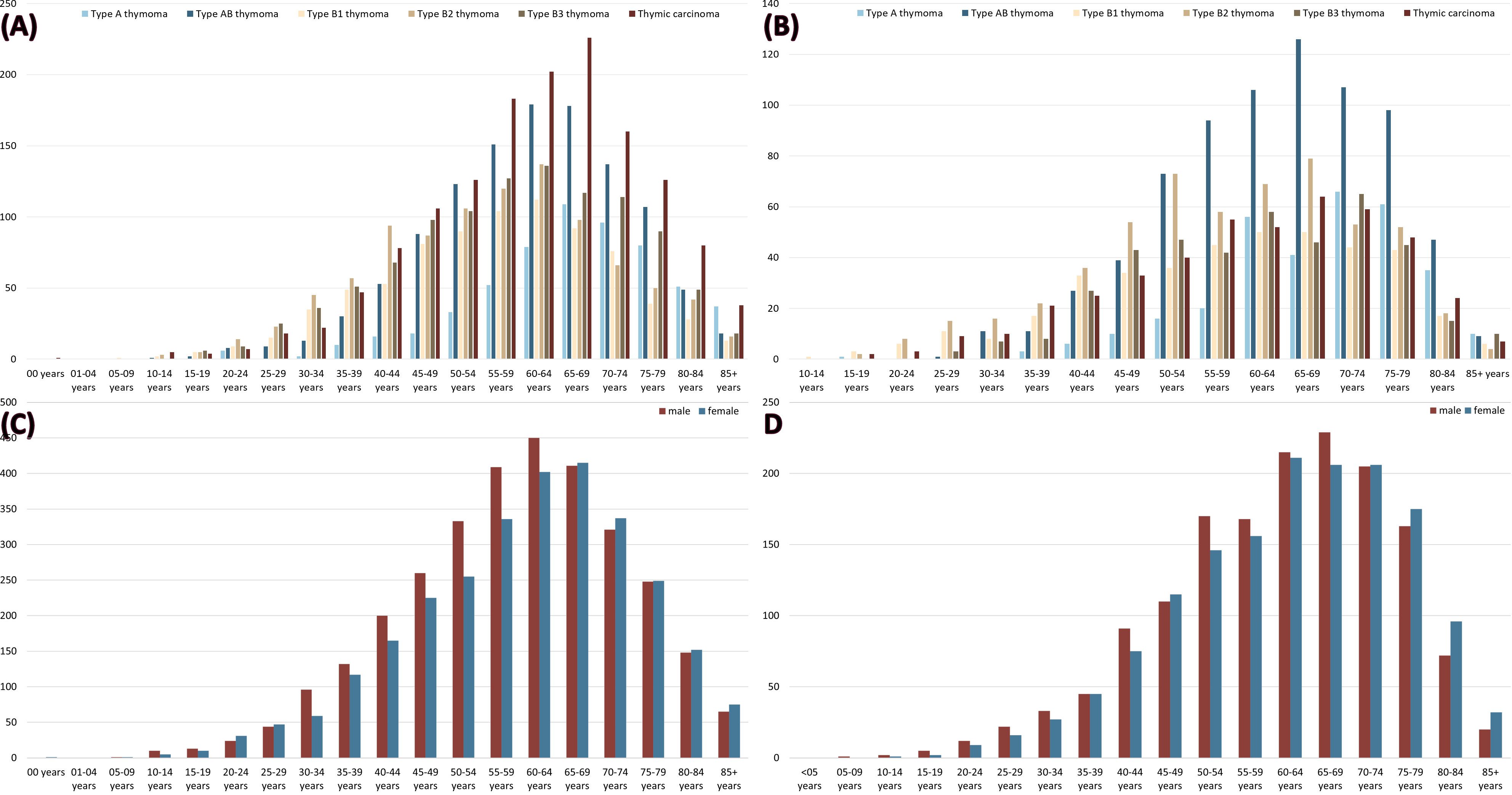

Supplement: Supplementary Figure 1 — (A) Age distribution of thymomas and thymic carcinomas in the (A) US from 2000-2019 and (B) Germany from 1999-2019. (B) Age and sex distribution of all epithelial tumors of the thymus in the (C) US from 2000-2019 and (D) Germany from 1999-2019. [file Image_1.tif]

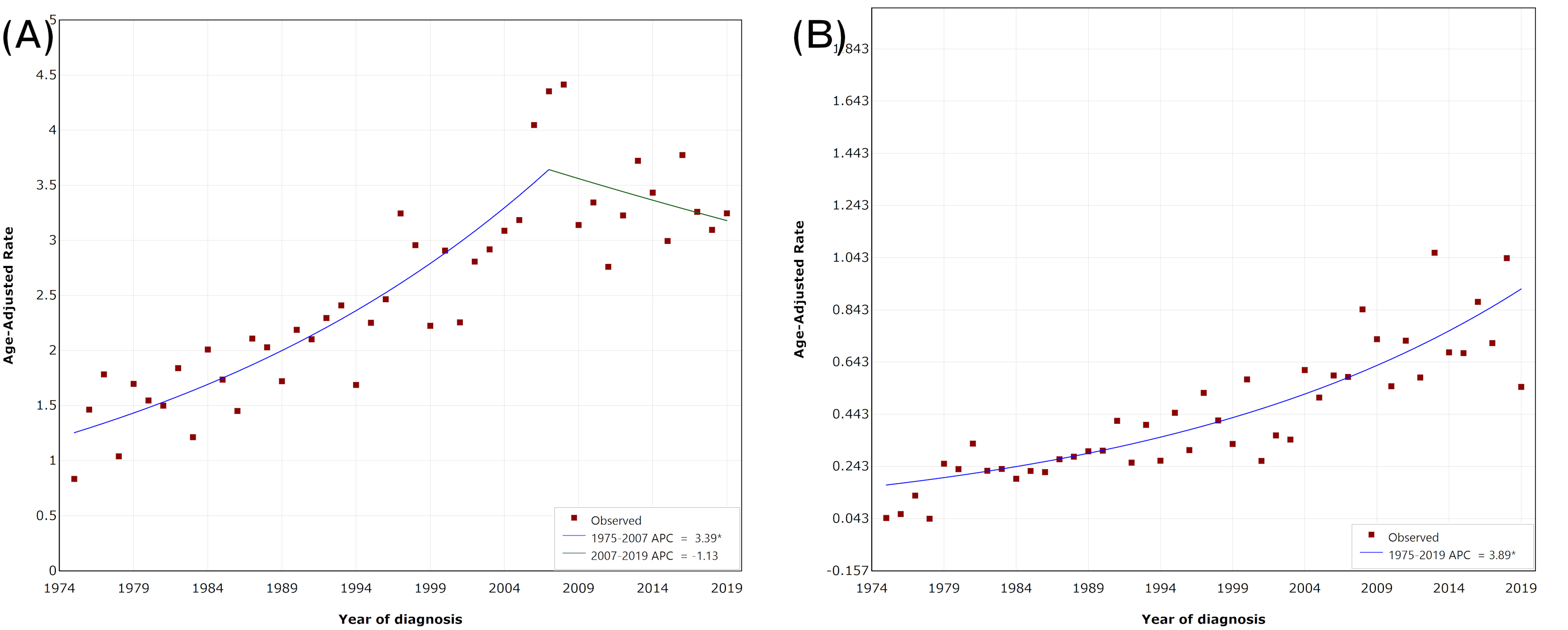

Supplement: Supplementary Figure 2 — Regression analysis of age-adjusted incidence rates per million between 1975 and 2019 of (A) all tumors of the thymus regardless of histology and (B) of all tumors of the thymus excluding epithelial tumors of the thymus, which are mostly comprised of tumors classified as squamous cell carcinomas (n=746), adenocarcinomas (n=502), and unspecified neoplasms (n=103). [file Image_2.tif]

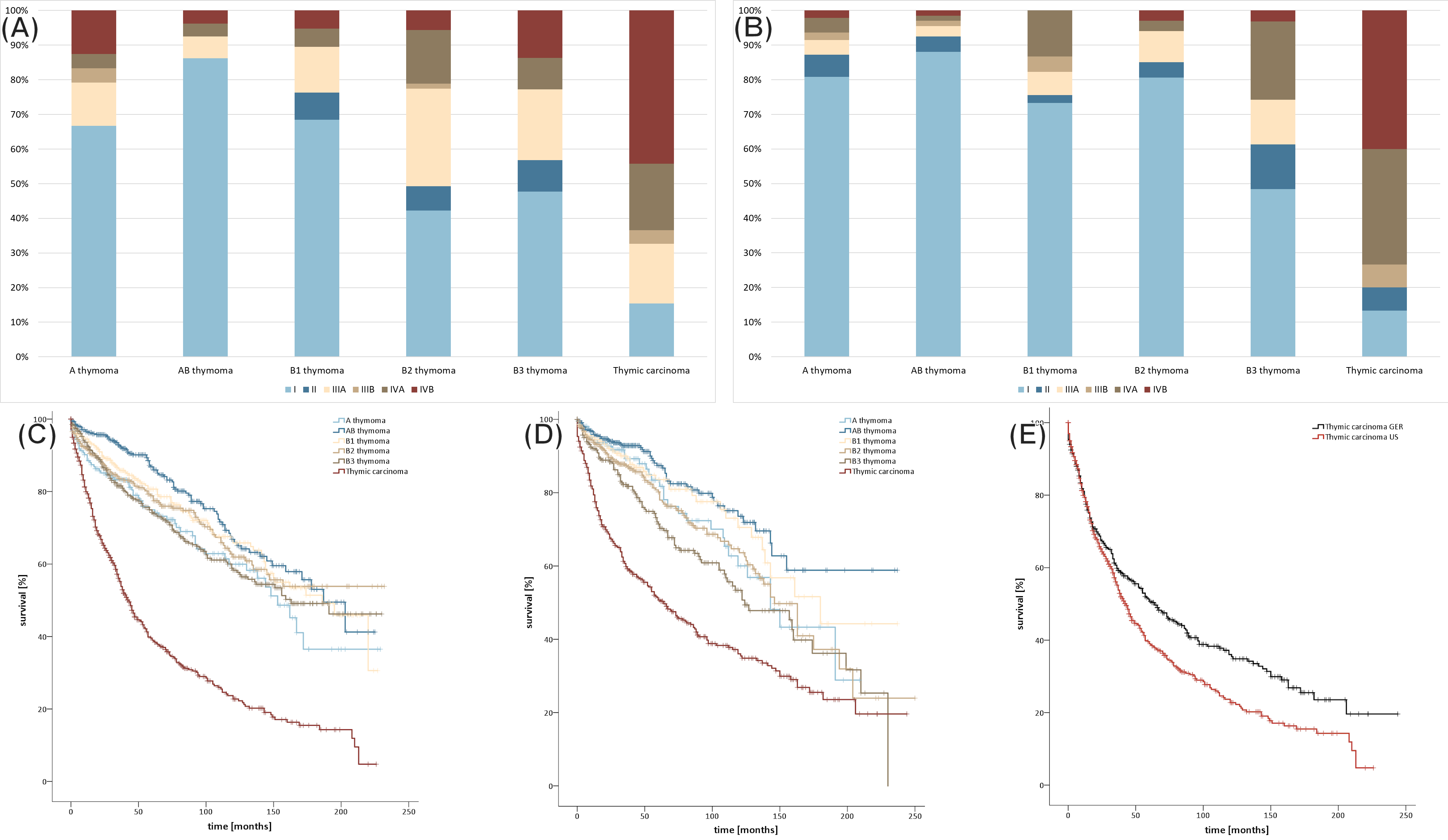

Supplement: Supplementary Figure 3 — Stage distribution of thymomas and thymic carcinomas in the (A) US from 2018-2019 and (B) Germany from 2017-2019. Survival stratified by subtype in the (C) US from 2000-2019 and (D) Germany from 1999-2019. (E) Significantly different survival of thymic carcinomas between the US and Germany. [file Image_3.tif]
